# Morphological and Molecular Characteristics of Perineuronal Nets in the Human Prefrontal Cortex—A Possible Link to Microcircuitry Specialization

**DOI:** 10.1007/s12035-024-04306-1

**Published:** 2024-07-03

**Authors:** Ivan Banovac, Matija Vid Prkačin, Ivona Kirchbaum, Sara Trnski-Levak, Mihaela Bobić-Rasonja, Goran Sedmak, Zdravko Petanjek, Natasa Jovanov-Milosevic

**Affiliations:** 1 Department of Anatomy and Clinical Anatomy, University of Zagreb School of Medicine, Šalata 11, HR-10000 Zagreb, Croatia; 2 Croatian Institute for Brain Research, Scientific Centre of Excellence for Basic, Clinical and Translational Neuroscience, School of Medicine University of Zagreb, Šalata 12, HR-10000 Zagreb, Croatia; 3 Department of Biology, University of Zagreb School of Medicine, Šalata 3, HR-10000 Zagreb, Croatia

**Keywords:** Extracellular matrix, Interneurons, Neurocan, Versican, GABA

## Abstract

Perineuronal nets (PNNs) are a type of extracellular matrix (ECM) that play a significant role in synaptic activity and plasticity of interneurons in health and disease. We researched PNNs’ regional and laminar representation and molecular composition using immunohistochemistry and transcriptome analysis of Brodmann areas (BA) 9, 14r, and 24 in 25 human postmortem brains aged 13–82 years. The numbers of VCAN- and NCAN-expressing PNNs, relative to the total number of neurons, were highest in cortical layers I and VI while WFA-binding (WFA^+^) PNNs were most abundant in layers III–V. The ECM glycosylation pattern was the most pronounced regional difference, shown by a significantly lower proportion of WFA^+^ PNNs in BA24 (3.27 ± 0.69%) compared to BA9 (6.32 ± 1.73%; *P* = 0.0449) and BA14 (5.64 ± 0.71%; *P* = 0.0278). The transcriptome of late developmental and mature stages revealed a relatively stable expression of PNN-related transcripts (log2-transformed expression values: 6.5–8.5 for VCAN and 8.0–9.5 for NCAN). Finally, we propose a classification of PNNs that envelop GABAergic neurons in the human cortex. The significant differences in PNNs’ morphology, distribution, and molecular composition strongly suggest an involvement of PNNs in specifying distinct microcircuits in particular cortical regions and layers.

**Supplementary Information:**

The online version contains supplementary material available at 10.1007/s12035-024-04306-1.

## Introduction

The neural extracellular matrix (ECM) is an essential component of the mammalian brain, making up to 20% of the mature brain volume [1–3]. In humans, the prenatally produced highly hydrated ECM begins the process of condensation perinatally, displaying fully developed condensed structures in some cortical regions as early as the 3^rd^ postnatal week [4, 5]. In the mature brain, ECM is mainly condensed around the soma, the dendrites’ proximal segments, and the axon initial segment (AIS) of neuronal subpopulations in the form of perineuronal nets (PNNs) [6–8]. The brain ECM is quite distinctive and is composed of hyaluronan, lecticans/hyalectans—a family of proteoglycans (PG) that includes aggrecan (ACAN), neurocan (NCAN), versican (VCAN), and brevican (BCAN)—and glycoproteins, such as tenascins and link proteins, that bind to the lecticans and hyaluronan [6, 7].

ACAN is the only lectican studied in the adult human cerebral cortex, where five clusters of cortical ACAN isoforms were identified in Brodmann area (BA) 6 [9]. NCAN is mainly associated with circuitry formation, synaptic plasticity critical period closure, and upregulated expression after various brain injuries [10, 11]. VCAN is found in at least four distinct isoforms (V0, V1, V2, and V3) [12–14], which show differential expression during development in the marginal zone and subplate [15] or in the hippocampal formation in the mature brain [16]. BCAN is present in two forms: a GPI-anchored form related to diffusely scattered glial cells and glial scars in the white matter and a soluble form found in the cortex where it transiently upregulates expression in learning and during the consolidation of memories [17–19].

In addition to having different core proteins, the lecticans mentioned above also differ in the amount and variety of glycosaminoglycan (GAG) side chains containing either *N*-acetylglucosamine or *N*-acetylgalactosamine. Among the enzymes responsible for glycosylation of brain PG are the 17 members of the *N*-acetylgalactosaminyltransferase (GALNT) family. *GALNT13* is particularly highly expressed in the cerebral cortex and cerebellum while low in other tissues [20, 21]. The *N*-acetylgalactosamine glycosylation of brain ECM in PNNs was successfully researched and visualized in histological slices of several mammalian species by different lectins, but most frequently using *Wisteria floribunda* agglutinin (WFA) [22–25].

It was determined that PNNs functionally mediate stabilization of the neuronal circuitry, either by selective regulation of new synapse formation as found in mouse neuron-astrocyte cultures [7, 8, 26–28] or by enhancement of the excitability of PV^+^ fast-spiking cortical neurons as seen in acute mouse brain slice cultures [29]. Research on *Gclm* KO mice and transgenic Tg2576 mice suggested the effects of ECM on synaptic stability as a neuroprotective mechanism in neurodegenerative diseases and oxidative stress in the anterior cingulate cortex [30, 31]. In that way, PNNs play a crucial role in neuronal homeostasis and are involved in plasticity, learning, and memory [32].

It is still inconclusive whether ECM-mediated synaptic stabilization is detrimental to recovery in models after CNS injury [33]. Models utilizing hyaluronidase that dissolves PNNs showed improved neuronal plasticity, axon regeneration, and functional recovery in animal models, such as mice and gerbils [34, 35]. However, hyaluronidase inhibition was also explicitly demonstrated to accelerate functional recovery from stroke in mouse models [36].

Previous research on ECM in the adult human brain mainly assessed the co-localization of PNNs with parvalbumin-expressing (PV^+^) GABAergic interneurons and visualized PNNs using WFA in postmortem brain tissue aiming to compare the brains of healthy individuals with those of individuals with specific brain pathologies. These studies reported findings of PNN reduction, changes in glycosylation pattern, and altered proteoglycan expression in schizophrenia, epilepsy, and Alzheimer’s disease [16, 23, 24, 37–42]. Except for ACAN, the expression of different types of lecticans as constituents of PNNs has not been studied in the healthy human brain [9]. Few studies utilized double- or triple-labeling immunofluorescence to evaluate the extent of co-expression of different ECM markers, except for ACAN and WFA [43, 44]. Data on the co-localization of different ECM and interneuron markers in the human brain are also scarce. In addition, spatiotemporal transcriptomic data for the expression pattern of PNN-related genes in the human brain are limited.

The literature review shows that the laminar distribution and morphological and molecular features of PNNs in the human brain have not been extensively studied. This points towards a need for more comprehensive systematic studies assessing different ECM markers in normotypical tissue using complementary histological methods. Therefore, this study aims to explore molecular composition, morphological features, and regional and laminar distribution of PNNs in the human prefrontal cortex (PFC). In particular, the study aims to comprehensively describe the variety and diversity of PNNs in phylogenetically and functionally distinct cortical areas (BA 9, 14r, and 24) using double and triple immunofluorescent labeling of different PNN constituents.

## Materials and Methods

### Brain Tissue Samples

Brain samples of five male human subjects aged 37 to 51 years with a postmortem delay of 6 to 11 h were analyzed using immunofluorescence (Table S1). The subjects had no medical history of neurological or psychiatric disorders and no neuropathological or signs of a preagonal state at autopsy. The brain tissue is stored in the brain bank as a part of the Zagreb Neuroembryological Collection [45, 46].

The University of Zagreb School of Medicine Ethics Committee approved the tissue collection and research conduction (approvals no. 380-59-10106-14-55/152 and 380-59-10106-19-111/210). The information on the subject’s identity and history is anonymized, and the brain samples are coded, indicating only the cortical region and the subject’s age.

The brain tissue blocks were cut according to Talairach’s coordinates [47]. Tissue blocks included the dorsolateral and ventromedial prefrontal cortex (dlPFC and vmPFC) containing the superior frontal gyrus (Brodmann area 9, BA9), straight gyrus (Brodmann area 14r, BA14r), and anterior cingulate cortex (Brodmann area 24, BA24) [48–50]. The tissue was fixed by immersion in 4% paraformaldehyde for 24 h, then dehydrated in an ethanol cascade (70%, 96%, 100%), vitrificated in toluene, and finally embedded in paraffin [51]. The tissue was then cut on a microtome into 20-μm-thick coronal slices [52] and mounted on VitroGnost Plus Ultra adhesive microscope slides (BioGnost, Zagreb, Croatia). According to relevant literature, the cortical regions and layers were delineated on NeuN-immunolabeled sections [49, 50, 53–56].

### Immunofluorescence Labeling

Double-labeling and triple-labeling immunofluorescence were performed according to protocols for paraffin-embedded tissue [25, 57, 58]. Histological sections were photobleached for 48 h using an LED light source [59, 60] to reduce lipofuscin autofluorescence. The sections were deparaffinized, and heat antigen retrieval was performed using a citrate-based (pH 6.0) unmasking solution [61]. Protein blocking was done by incubating the sections for 1 h at room temperature (RT) in normal donkey serum (NDS; Chemicon, USA) or bovine serum albumin (BSA; Sigma-Aldrich, USA) diluted 1:30 in permeabilization solution (0.3% Triton X-100 in 1X PBS; Sigma-Aldrich, USA). Afterwards, the sections were incubated overnight in primary antibodies (Table S2) at 4 °C and in secondary antibodies (Table S2) for 2 h at RT. The sections were treated with TrueBlack Lipofuscin Autofluorescence Quencher (Biotium, USA) to reduce autofluorescence further [57, 62] and coverslipped with VectaMount Aqueous Mounting Medium (Vector Laboratories, USA). Anti-NeuN antibodies were combined with all ECM markers—WFA, anti-NCAN, and anti-VCAN (V0 isoform) antibodies. The ECM markers were also combined with different interneuron markers—anti-parvalbumin (PV), anti-calretinin (CR), and anti-somatostatin (SOM) antibodies. Finally, all three ECM markers were combined using triple labeling.

### Quantification of Immunoreactive Cells

Quantitative analysis of immunoreactive cells and condensed perisomatic ECM was done using Neurolucida 2020 (MBF Bioscience, Williston, Vermont, USA) and Neurolucida Explorer (MBF Bioscience) on confocal images of double-labeled immunofluorescent histological slides (NeuN/ECM markers and PV/WFA). NeuN immunolabeling was used to delineate the cortical layers. Contours from neighboring NeuN-labeled slides were used to determine cortical layers in PV/WFA double-labeled sections. For each marker combination (NeuN/WFA, NeuN/NCAN, NeuN/VCAN, and PV/WFA), three slides from each region of each brain specimen (*n* = 5) were quantified using the *Detect cells* function in the Neurolucida software, after which manual correction was performed by two independent observers [57].

Condensed perisomatic ECM was counted as a PNN only if it met all the following criteria: (1) condensed ECM surrounding a visible soma outline, (2) clear distinction between the perisomatic ECM and background staining was present due to a difference in staining intensity, (3) perisomatic ECM surrounded at least 50% of the soma’s outline, (4) condensed ECM enveloped at least the proximal part of the cellular processes. Condensed ECM surrounding visible cellular outlines that did not meet these criteria were counted as ECM aggregates. On NeuN/ECM markers double-labeled slides, only PNNs or ECM aggregates surrounding NeuN-immunolabeled (NeuN^+^) cells were counted.

NeuN/ECM markers quantification results are presented as (a) the proportion of NeuN^+^ cells surrounded by a specific PNN or ECM aggregate and (b) the proportion of total PNNs or ECM aggregates found in a specific cortical layer. The results of PV/WFA double-labeling quantification are presented as (a) the proportion of PV^+^ cells surrounded by WFA^+^ PNNs and (b) the proportion of WFA^+^ PNNs surrounding PV^+^ cells.

### Quantification of PNN Thickness

To determine the difference in the thickness of different types of PNNs, we measured the thickness of 20 representative PNNs for each type of PNN. For each PNN, three measurements were made from which the average value was taken. The PNN thickness was measured at its thinnest part, where the distinction between the PNN and background/cellular staining was the clearest.

### Statistical Analysis

The statistical analysis used GraphPad Prism, version 10.0.2 (GraphPad Software, La Jolla, USA).

Data are shown as arithmetic mean ± standard deviation (SD). Data from the same brain were analyzed as paired data. Repeated measures (RM) one-way ANOVA was used to test the differences between BA9, BA14r, and BA24. Tukey’s multiple comparison test was used to determine which exact comparisons were significantly different. A *P*-value of less than 0.05 was considered statistically significant, and 95% confidence intervals (CI) were provided for the mean differences.

### Transcriptome Analysis

Using previously published microarray database from healthy human brains [63] available by the Gene Expression Omnibus (GEO accession GSE 25219, Human Exon 1.0 ST Array), the mRNA expression patterns were analyzed for the following genes: *NCAN*, *VCAN*, *GALNT1*, *GALNT2*, *GALNT9*, *GALNT11*, *GALNT13*, *GALNT16*, and *GALNT17*. The gene expression was examined in three regions of interest: the dorsal frontal cortex (DFC), the medial frontal cortex (MFC), and the orbitofrontal cortex (OFC) containing BA9, BA24, and BA14r, respectively. The transcriptomic data are obtained from anatomically defined areas as specified in the supplementary figures’ descriptions (for details on the dissection protocol for transcriptomic data, see Kang et al. Nature 2011, supplementary material). Furthermore, the same person (G.S.) dissected both sample sets, tissue samples for transcriptomic data, and tissue blocks for immunohistochemical experiments in this study. Thus, we are confident that they both represent the equivalent anatomical areas. Twenty postmortem brains from 13 to 82 years of age (Table S1), totaling 98 samples from both the left and right hemispheres [63], were analyzed using Partek Genomic Suite 6.6. (Partek Inc.) to normalize data and summarize probe sets and transcript clusters. Affymetrix CEL files were imported into Partek Genomic Suite using default Partek settings, and the analysis was done on the probe sets designated as “core” and “extended” using Partek Genomic Suite 6.6 and R Statistical Software Package (http://www.r-project.org). The analyzed probe sets covered the entire span of the gene. The data are presented as the log2-transformed value of the signal intensity, and a gene was considered expressed when the log2-transformed expression value in the analyzed sample was ≥ 5.5 [63, 64].

## Results

The full-scale qualitative and quantitative analysis was applied to all three cortical regions. We explicitly stated in the text if regional differences were found during the study.

### Perineuronal Nets in the Human Prefrontal Cortex Can Be Classified into Distinct Morphological Types

For each ECM marker (WFA, VCAN (V0), and NCAN), we detailedly analyzed its PNN morphology in three cortical regions: BA9, BA14r, and BA24. We classified PNNs into distinct morphological types based on the following parameters: 2D shape, the extent of envelopment of cellular processes, thickness of the envelope, and the staining intensity (Table 1). We determined that certain morphological types of PNNs were found only in specific cortical layers.

**Table 1:** Morphological classification of different perineuronal nets

PNN molecular constituent	PNN type	Staining intensity	Morphological shape	Envelops processes beyond the first branching point	Thickness (μm)	Cortical layers in which PNNs were found
WFA	1	+++	Circular	Yes	3.49 ± 0.82	II–VI
	2	++	Circular	No	2.20 ± 0.20	II–VI
	3	++	Pyramidal	No	2.41 ± 0.34	III, V
VCAN	1	+++	Fusiform	No	4.37 ± 1.04	VIb, WM, occasionally VIa
	2	+/++	Circular	No	2.68 ± 0.41	I–VI (predominantly IV)
	3	++	Pyramidal	No	2.35 ± 0.30	Sporadically V, VI
NCAN		++/+++	Circular	No	2.74 ± 0.53	I–VI

**Fig. 1:**
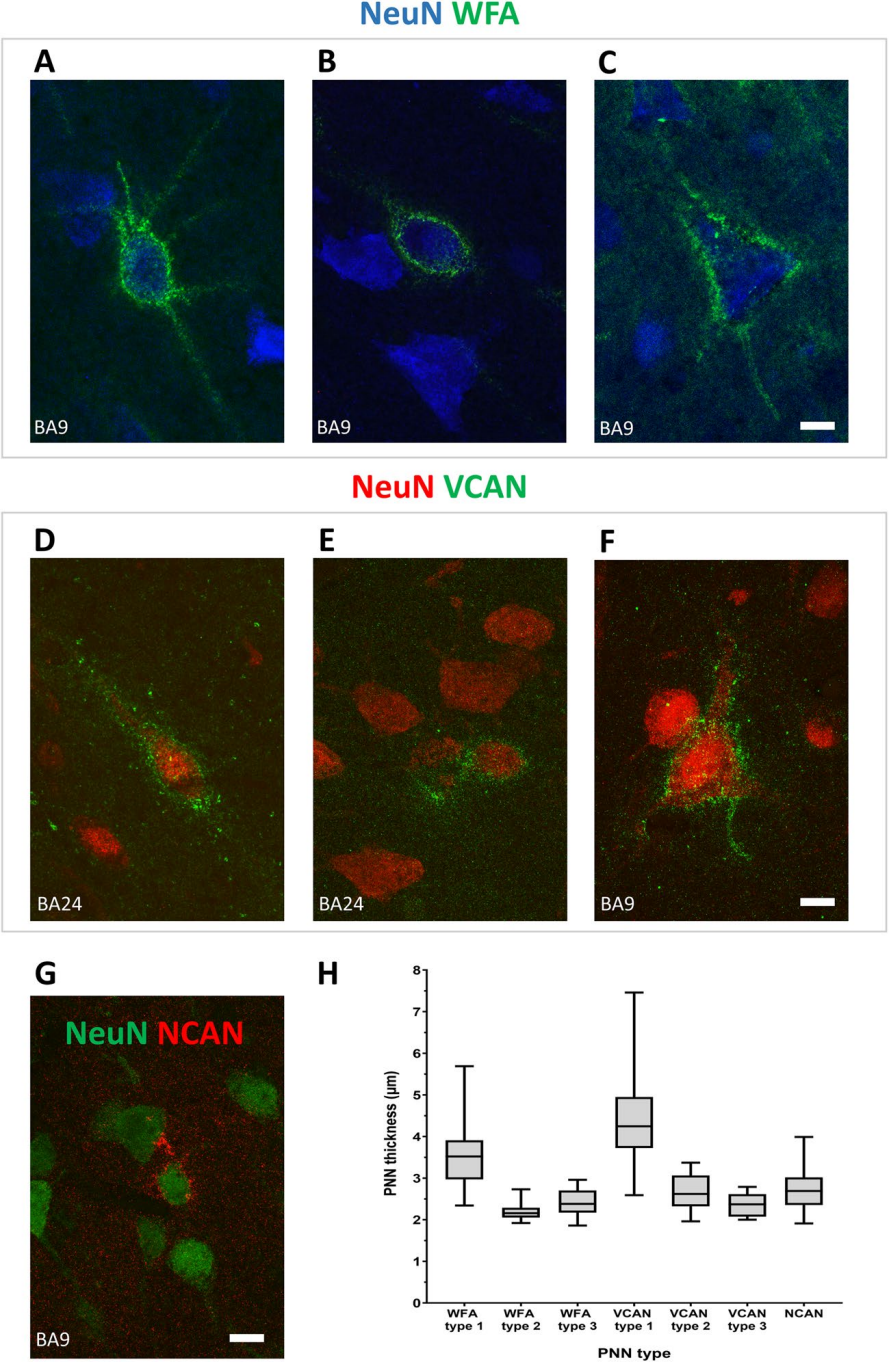
Morphological types of PNNs in the human PFC. **A** WFA^+^ type 1 PNN surrounding a circular NeuN^+^ cell in layer III of BA9. This PNN type envelops the cell body and the cellular processes beyond the first branching point. **B** WFA^+^ type 2 PNN surrounding a circular NeuN^+^ cell in layer III of BA9. This PNN type is thinner and envelops only the cell body and the most proximal parts of the cellular processes before the first branching point. **C** WFA^+^ type 3 PNN surrounding a pyramidal NeuN^+^ cell in layer III of BA9. This PNN type, similar to type 2, envelops only the cell body and the most proximal parts of the cellular processes. **D** VCAN^+^ type 1 PNN surrounding a fusiform NeuN^+^ cell in layer VI of BA24. As with all VCAN^+^ PNNs, the PNN envelops only the cell body and the most proximal parts of the cellular processes. This PNN type is the thickest type of VCAN^+^ PNNs and is present predominantly in the infragranular cortical layers. **E** VCAN^+^ type 2 PNN surrounding a circular NeuN^+^ cell in layer III of BA24. This PNN type is thinner and present predominantly in the granular and supragranular cortical layers. **F** VCAN^+^ type 3 PNN surrounding a pyramidal NeuN^+^ cell in layer V of BA9. This PNN type was rather scarce and found only in infragranular layers. **G** NCAN^+^ PNN in layer III of BA9. NCAN^+^ PNNs were thin and enveloped only the cell body and the most proximal parts of the cellular processes. Scale bar for all panels: 10 μm. **H** Box plots displaying the measured thickness of different types of PNNs. Error bars represent the maximum and minimum values

Our analysis revealed that at least three types of WFA^+^ PNNs could be differentiated. Type 1 WFA^+^ PNNs appear circular, surround ovoid neuron’s soma, have a high level of staining intensity, and envelop the cellular processes beyond the first branching point (Fig. 1A). Type 2 WFA^+^ PNNs also surrounded ovoid neurons’ soma but had a comparatively lower level of staining intensity and typically did not envelop the cellular processes beyond the first branching point (Fig. 1B). Unlike types 1 and 2, type 3 WFA^+^ PNNs appear triangular and surround the pyramidal neurons (Fig. 1C). The other characteristics of type 3 WFA^+^ PNNs were similar to those of type 2. The 3D presentations of all types of PNNs (five moves) are provided in the Supplementary material: 3D reconstructions.

While type 1 and 2 WFA^+^ PNNs were found in layers II–VI, type 3 PNNs were found almost exclusively in layers III and V.

The analysis of VCAN^+^ PNNs, similar to WFA^+^ PNNs, revealed at least three morphological types. Type 1 VCAN^+^ PNNs surrounded larger fusiform neurons with high staining intensity (Fig. 1D). Type 2 VCAN^+^ PNNs surrounded smaller ovoid neurons with a comparatively lower staining intensity (Fig. 1E). While type 1 and 2 VCAN^+^ PNNs were relatively common, type 3 VCAN^+^ triangular PNNs were found only sporadically and surrounded pyramidal neurons (Fig. 1F). All three types of VCAN^+^ PNNs did not envelop the cellular processes beyond the first branching point. While type 2 VCAN^+^ PNNs were found in all cortical layers, type 1 VCAN^+^ PNNs were present predominantly in layer VIb and the superficial white matter. We found type 3 VCAN^+^ PNNs only in layers V and VI.

Unlike with WFA and VCAN, we could clearly distinguish only a single morphological type of NCAN^+^ PNNs. These PNNs were found in all cortical layers, surrounded smaller ovoid neurons, and did not envelop the cellular processes beyond the first branching point (Fig. 1G). The staining intensity of NCAN^+^ PNNs was similar to that of type 2 and 3 WFA^+^ PNNs.

The measured thickness of different types of PNNs was in line with their staining intensity (Table 1 and Fig. 1H).

Besides PNNs, we also identified condensed ECMs that did not meet the criteria for classification as PNNs. We defined such structures as ECM aggregates. Compared to PNNs, ECM aggregates had lower staining intensity, were not demarcated from the background, and did not envelop the cellular processes (Figure S1).

### Different PNN Molecular Constituents Have Specific Laminar Distributions

**Fig. 2:**
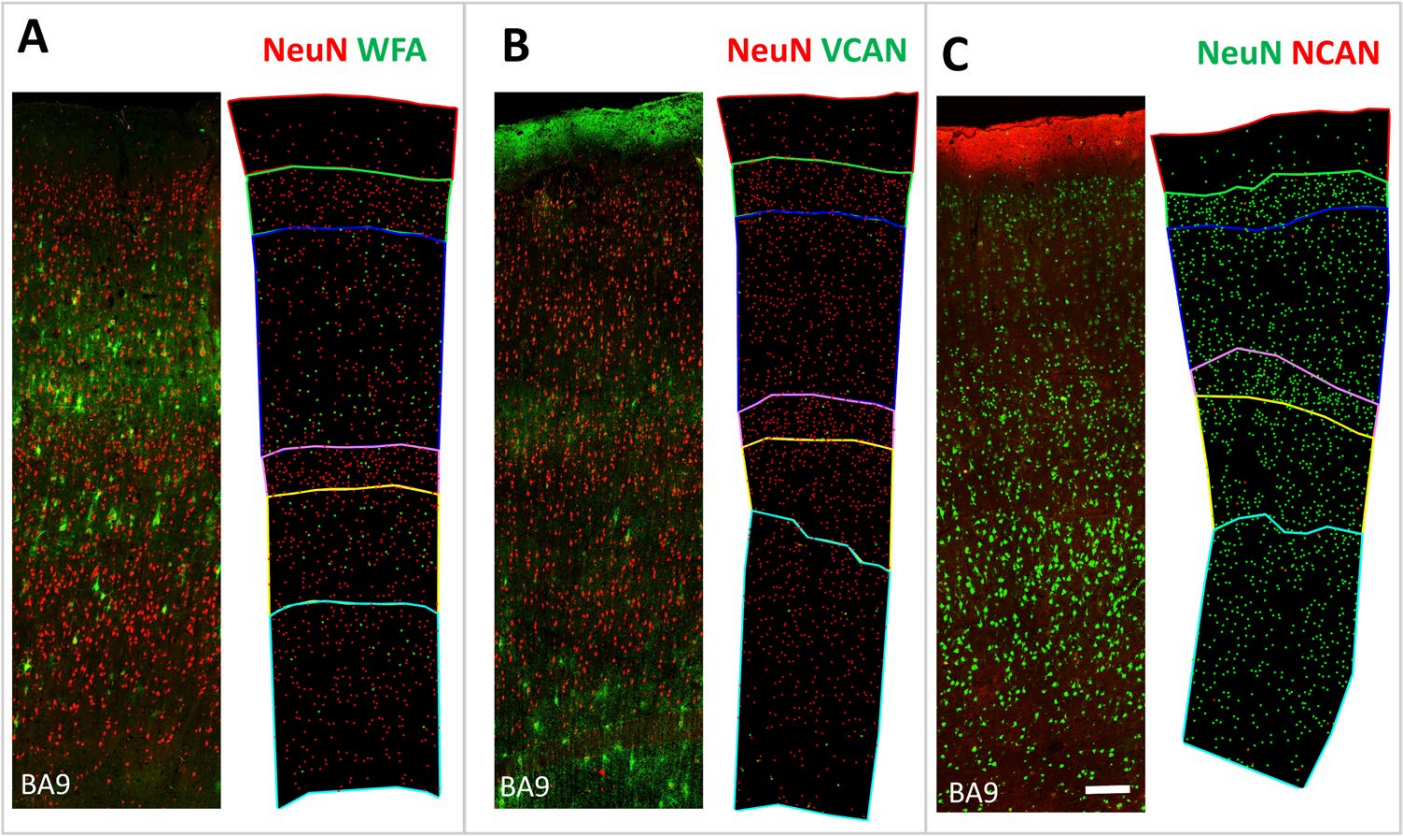
Laminar distribution of different types of ECM markers in the human PFC. **A** NeuN/WFA, **B** NeuN/VCAN, and **C** NeuN/NCAN double labeling in BA9 with corresponding dot plots showing the distribution of NeuN^+^ cells and PNNs in different cortical layers. It is visible that WFA^+^ PNNs were the most numerous and that they are present in all cortical layers, including sporadically in layer I. Most WFA^+^ PNNs were located in layers III and V. VCAN^+^ and NCAN^+^ PNNs were less numerous and mostly located in layer VI. Scale bar: 100 μm

**Fig. 3:**
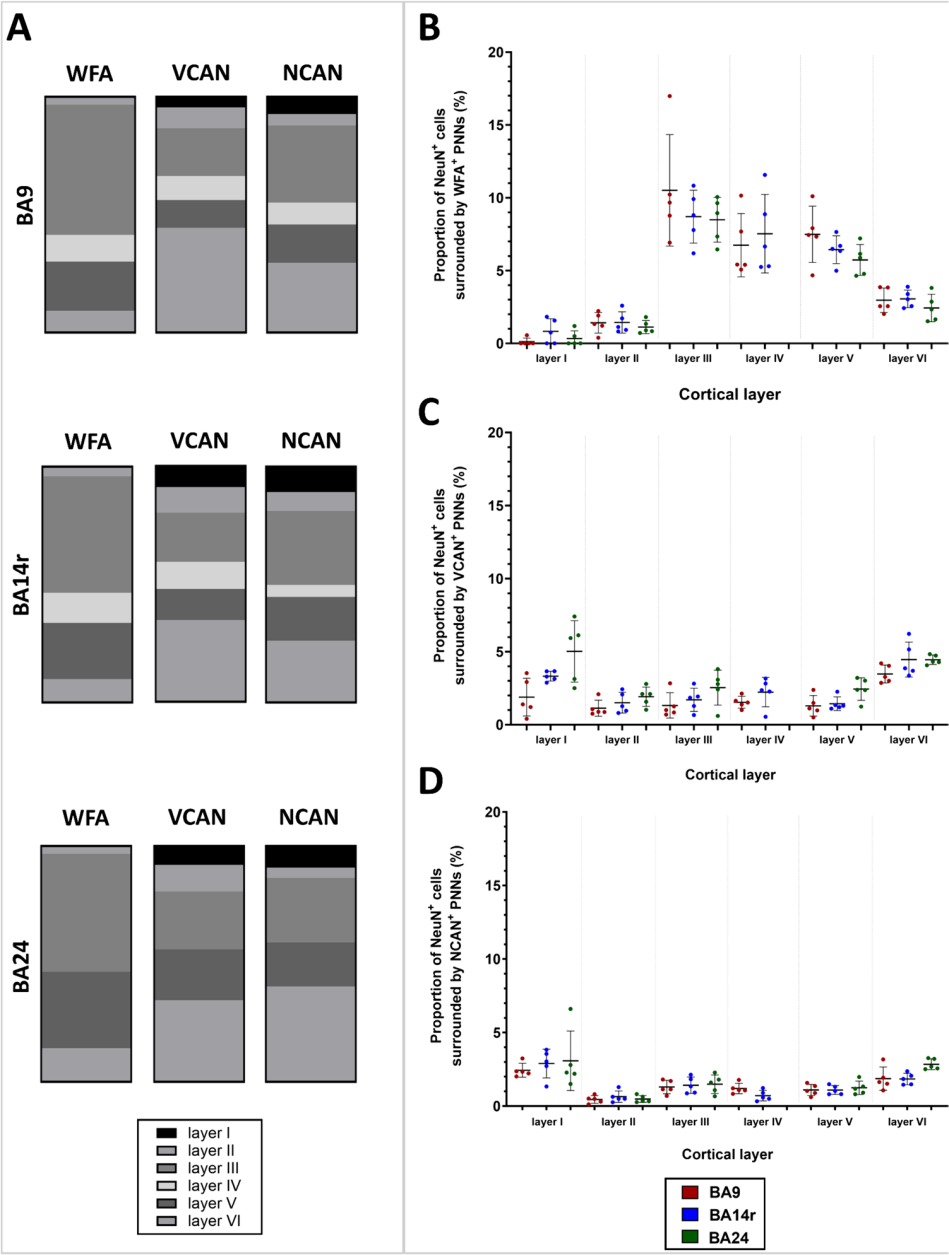
Laminar distribution of different types of PNNs in the human PFC. **A** Bar graphs represent the proportion of PNNs in specific cortical layers in relation to the total number of PNNs in the cortex. Note the specific laminar distribution patterns of each ECM constituent as well as the specific distributions in BA24 reflective of the cytoarchitectonic structure of this region. Scatter plots showing the proportion of NeuN^+^ neurons surrounded by **B** WFA^+^ PNNs, **C** VCAN^+^ PNNs, and **D** NCAN^+^ PNNs in different cortical layers. Data are presented as mean ± SD. Note that almost 10% of cortical neurons in layers III, IV, and V were surrounded by WFA^+^ PNNs, while up to 5% of cortical neurons in layers I and VI were surrounded by either VCAN^+^ or NCAN^+^ PNNs

For each ECM molecular marker, we quantified the number of PNNs and the number of condensed ECM aggregates in all cortical layers in all three cortical regions (BA9, BA14r, and BA24). Our analysis revealed that each ECM constituent had a specific laminar distribution (Figs. 2 and 3).

We first analyzed the proportion of PNNs in specific cortical layers in relation to the total number of PNNs in the cortex. Out of all WFA^+^ PNNs, over 50% were found in layer III, and over 20% were found in layer V. Up to 10% of WFA+ PNNs were found in layers II, IV, and VI each. Interestingly, we also found WFA^+^ PNNs sporadically in layer I (Figure S2). In contrast, around 40% of all VCAN^+^ PNNs were found in layer VI, while over 20% were found in layer III. Up to 10% of VCAN^+^ PNNs were found in the other cortical layers. Similar to VCAN^+^ PNNs, most NCAN^+^ PNNs were found in layers III and VI—around 30% in each layer—while around 18% were found in layer V. Between 5 and 10% of NCAN^+^ PNNs were found in layers I, II, and IV (Fig. 3A).

We then analyzed the proportion of neurons surrounded by PNNs in different cortical layers. The proportion of neurons surrounded by WFA^+^ PNNs was the highest in layer III, where WFA^+^ PNNs surrounded, on average, 10% of the neurons in that cortical layer. WFA^+^ PNNs surrounded 7% of neurons in layers IV and V. The proportion of neurons surrounded by WFA^+^ PNNs was far lower in cortical layers I, II, and VI (Fig. 3B). The proportion of neurons surrounded by VCAN^+^ PNNs was the highest in layers I and VI, where VCAN^+^ PNNs surrounded up to 5% of the neurons in those cortical layers. The proportion of neurons surrounded by VCAN^+^ PNNs was uniformly low in all other cortical layers (Fig. 3C). The proportion of neurons surrounded by NCAN^+^ PNNs was the highest in layer I, where NCAN^+^ PNNs surrounded up to 4% of the neurons in that cortical layer. The proportion of NCAN^+^ PNNs was uniformly lower in all other cortical layers (Fig. 3D).

Analysis of ECM aggregates revealed that WFA^+^ aggregates were most numerous in layer III (Figure S3A), while VCAN^+^ aggregates were most numerous in layer I (Figure S3B). NCAN^+^ aggregates were relatively uniformly distributed across all cortical layers (Figure S3C).

### PNNs Enveloped Each GABAergic Interneuron Population with a Distinct Molecular Composition

For each ECM marker, we evaluated which population of interneurons (PV, CR, and SOM) it co-localized with (Table 2). We found no qualitative differences between the three analyzed regions (BA9, BA14r, and BA24).

**Table 2:** Co-localization of different PNN types with different interneuron markers in all analyzed cortical regions

ECM marker	PNN type	Co-localization		
		PV	CR	SOM
WFA	1	Yes (layers II–VI)	No	Sporadically layer V/VI
	2	Yes (layers II–VI)	No	No
	3	No	No	No
VCAN	1	No	No	No
	2	No	Yes (layers I, III, and IV)	Yes (layers V/VI)
	3	No	No	No
NCAN		Yes (layer III)	Yes (layers I, V/VI)	Yes (layers V/VI)

**Fig. 4:**
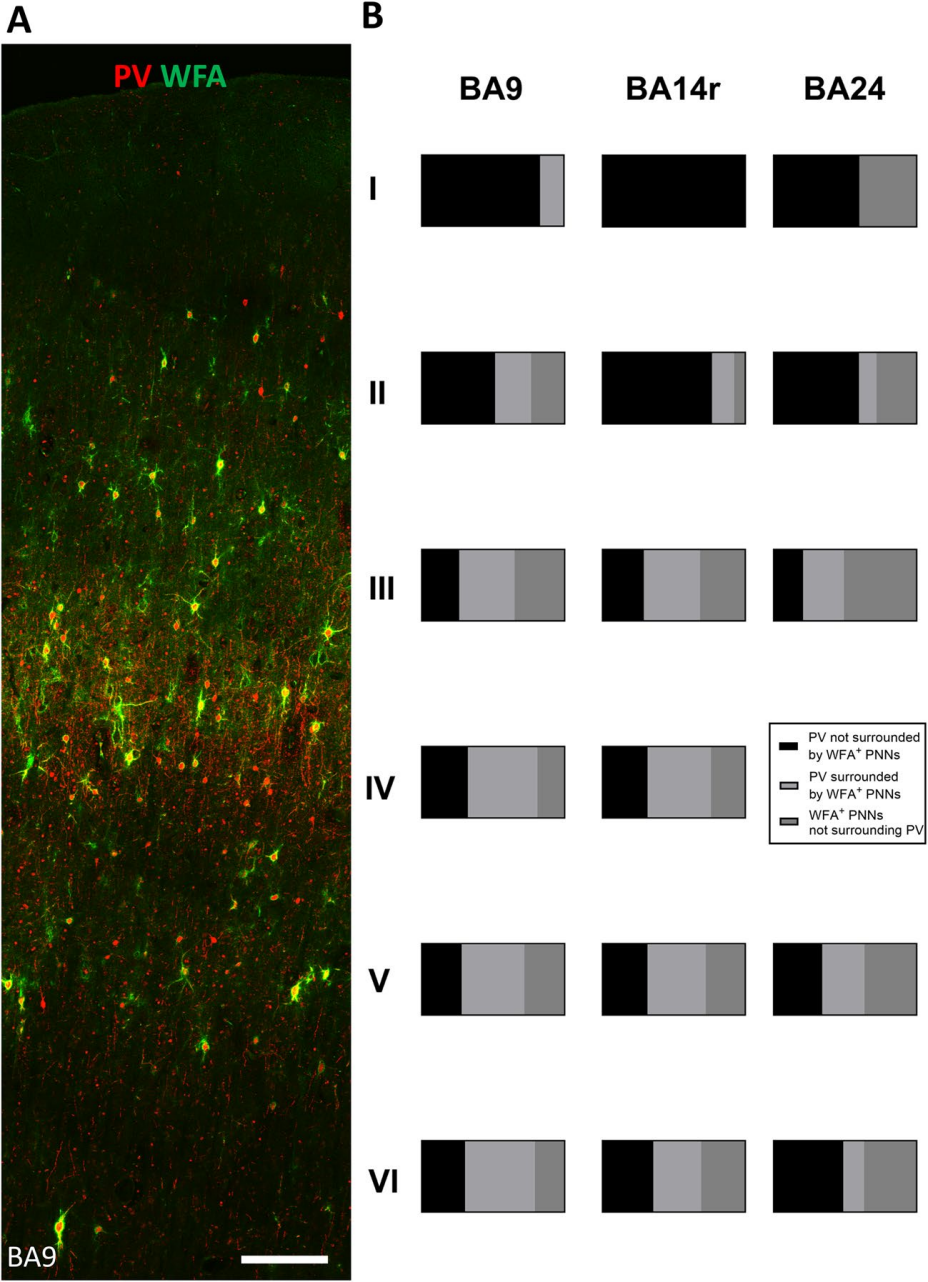
Co-localization of PV and WFA in the human PFC. **A** PV/WFA double labeling, BA9. Scale bar: 50 μm. It is evident that WFA^+^ PNNs enveloped a substantial number of PV^+^ neurons. However, there were also PV^+^ neurons not surrounded by WFA^+^ PNNs and WFA^+^ PNNs that did not envelop PV^+^ neurons. **B** Bar graphs represent the relative proportions of cells expressing only PV, cells only surrounded by WFA^+^ PNNs, and cells expressing PV and surrounded by WFA^+^ PNNs. Note the layer-specific relative proportions as well as the regional differences, which were most pronounced in layers II and VI

We confirmed that type 3 WFA^+^ PNNs and VCAN^+^ PNNs did not surround any of the cells among the major interneuron populations. Type 1 and 2 WFA^+^ PNNs almost exclusively surrounded PV^+^ neurons (Fig. 4A).

Since the PV and WFA co-expression levels were rather high, we quantitatively analyzed the co-expression levels in different cortical layers. The proportion of PV^+^ neurons surrounded by WFA^+^ PNNs was the highest in layers III, IV, and V. A similar pattern was determined for the proportion of WFA^+^ PNNs surrounding PV^+^ neurons (Fig. 4B). Among the population of cells that either expressed PV or were surrounded by WFA^+^ PNNs, three subpopulations could be defined: (1) cells expressing only PV, (2) cells only surrounded by WFA^+^ PNNs, and (3) cells both expressing PV and surrounded by WFA^+^ PNNs. We determined that the relative proportions of these three subpopulations were highly layer-specific, with layers II and VI suggesting regional specificity (Fig. 4B).

**Fig. 5:**
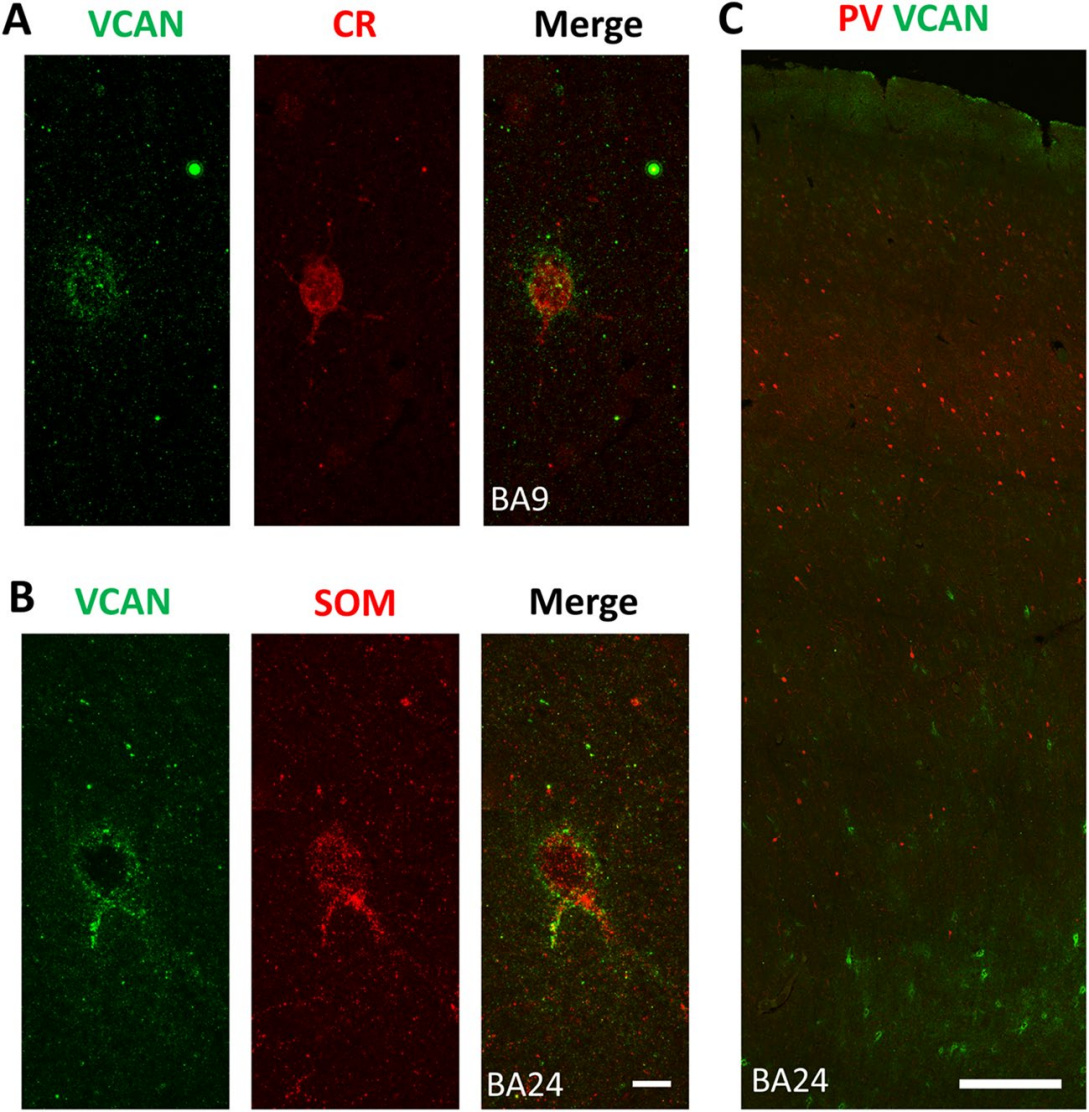
Co-localization of VCAN with different interneuron markers in the human PFC. **A** CR/VCAN double labeling in layer III of BA9, and **B** SOM/VCAN double labeling in layer V of BA24, showing co-localization. Scale bar for both panels: 10 μm. **C** PV/VCAN double labeling in BA24 showing no co-localization. Scale bar: 100 μm

Type 1 VCAN^+^ PNNs were not typically found surrounding any of the three interneuron populations. In contrast, type 2 VCAN^+^ PNNs surrounded CR^+^ neurons in layers I, III, and IV (Fig. 5A) and SOM^+^ neurons in layers V/VI (Fig. 5B). We did not observe co-localization between VCAN and PV (Fig. 5C).

**Fig. 6:**
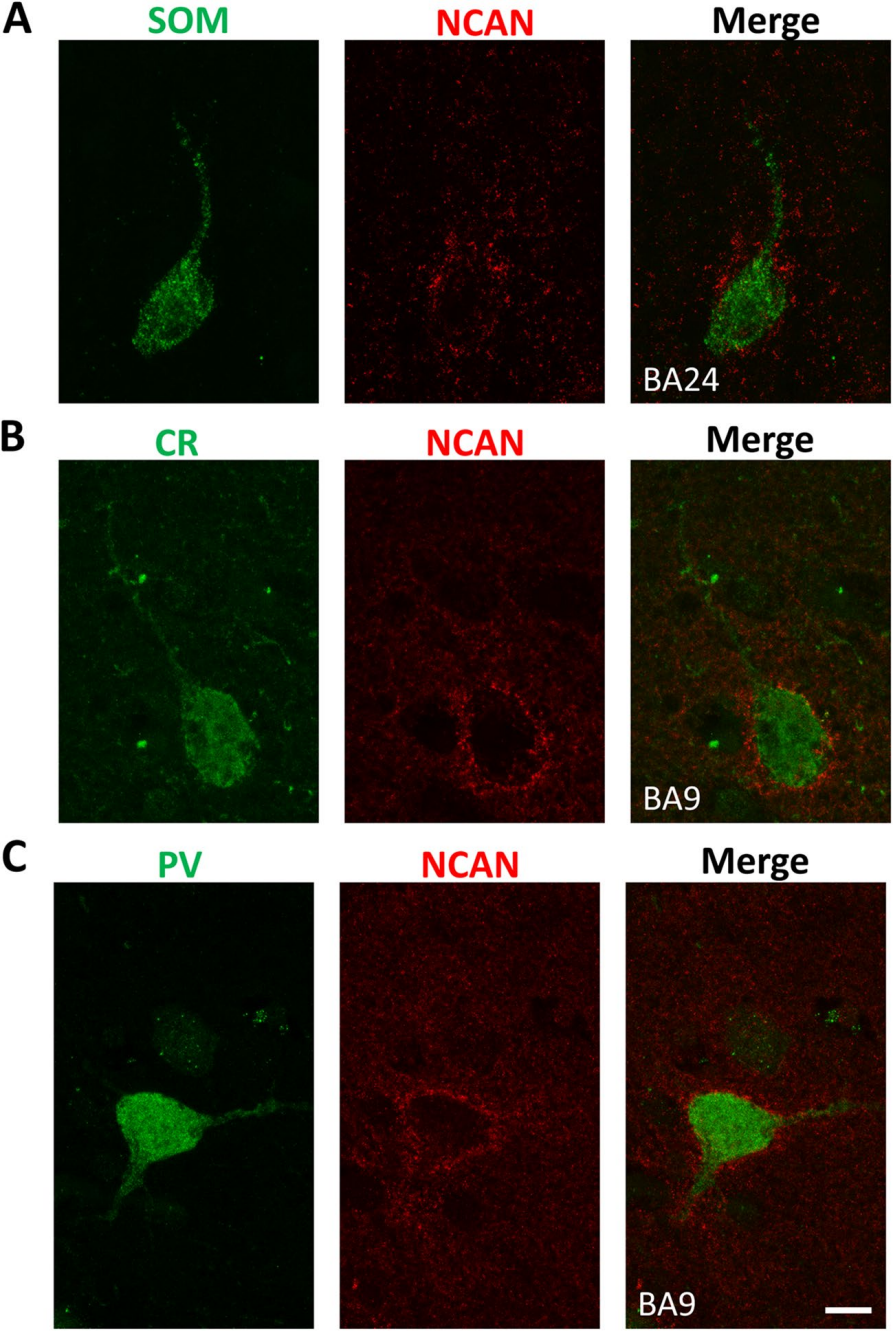
Co-localization of NCAN with different interneuron markers in the human PFC. **A** SOM/NCAN double labeling in layer V of BA24, **B** CR/NCAN double labeling in layer V of BA9, and **C** PV/NCAN double labeling in layer III of BA9 showing co-localization. Scale bar for all panels: 10 μm

NCAN^+^ PNNs surrounded cells from all three interneuron populations; however, in layer I, they were found primarily around CR^+^ neurons, in layers V and VI around both SOM^+^ (Fig. 6A) and CR^+^ neurons (Fig. 6B) and in layer III around PV^+^ neurons (Fig. 6C).

### Co-expression of WFA, VCAN, and NCAN Is Relatively Low in the Human PFC

**Fig. 7:**
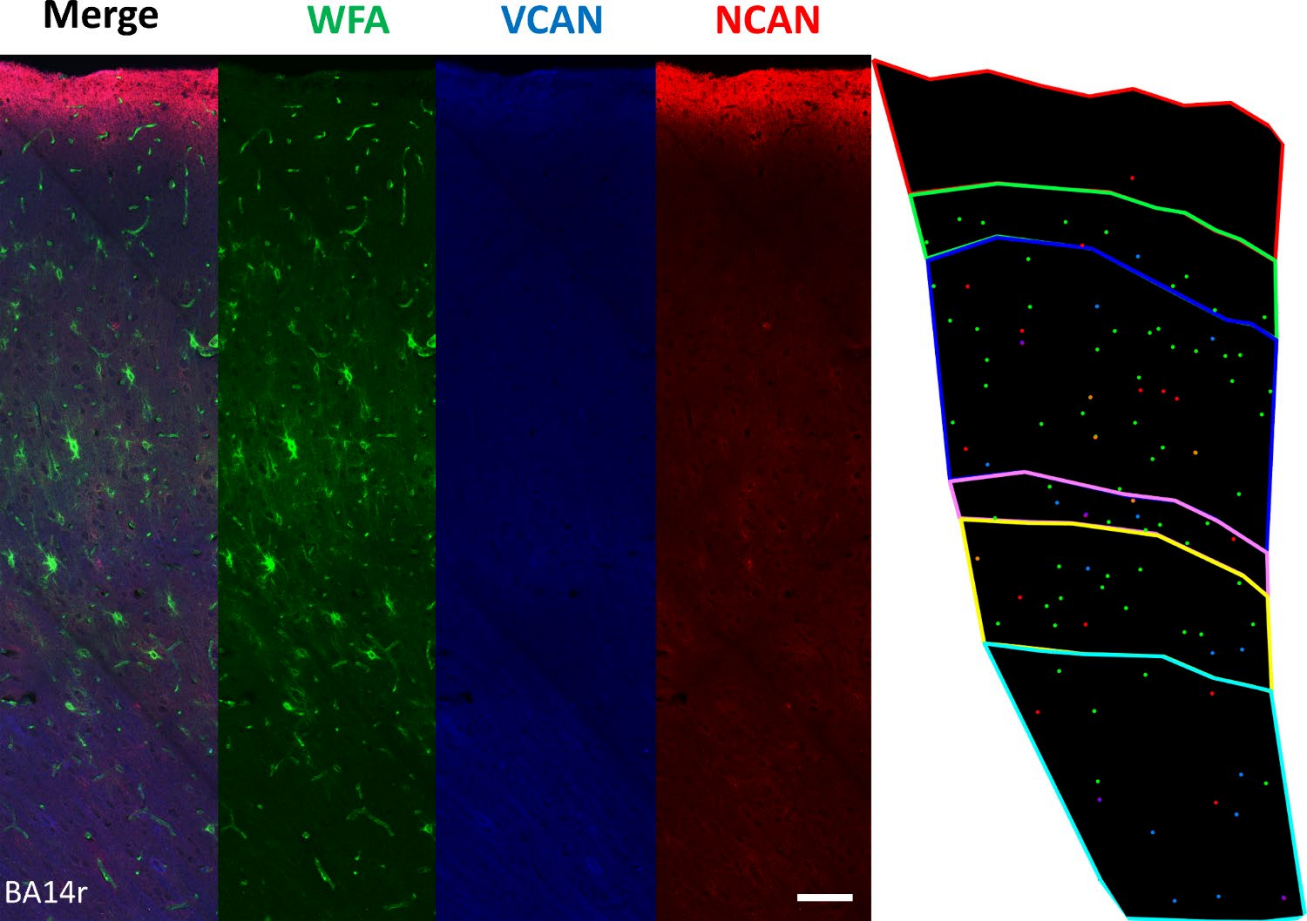
WFA/VCAN/NCAN triple-labeling immunofluorescence in BA14r with corresponding dot plot. Green markers on the dot plot represent WFA^+^ PNNs, blue markers represent VCAN^+^ PNNs, and red markers represent NCAN^+^ PNNs. Orange markers represent WFA/NCAN co-localization, while purple markers represent VCAN/NCAN co-localization. No WFA/VCAN co-localization was found. Scale bar: 100 μm

In order to determine the co-expression of different ECM markers, we performed WFA/NCAN/VCAN triple labeling in all three cortical regions (BA9, BA14r, and BA24). We determined that most cells seemed surrounded by only one of the three analyzed ECM markers, i.e., the level of ECM marker co-localization was rather low overall. However, we identified PNNs with VCAN/NCAN co-localization and other PNNs with NCAN/WFA co-localization. Our analysis did not reveal any PNNs with VCAN/WFA co-localization or any PNNs with triple co-localization (Fig. 7).

### Regional Differences in WFA Expression Are More Pronounced than Differences in VCAN and NCAN Expression

Finally, we compared the expression of different ECM markers in the three analyzed regions of the cerebral cortex (BA9, BA14r, and BA24).

**Fig. 8:**
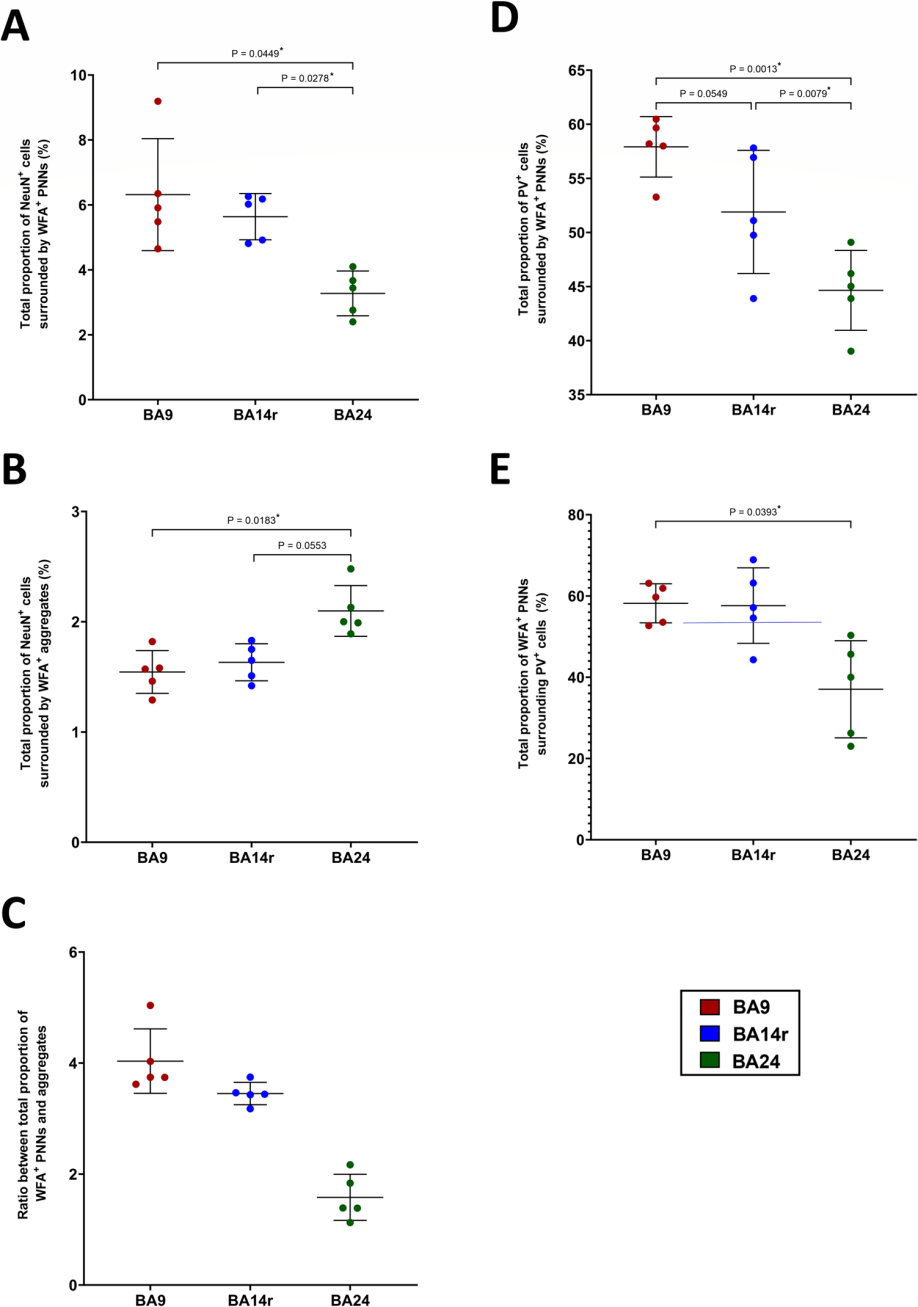
Regional differences in the expression of WFA. Scatter plots representing the **A** proportion of NeuN^+^ cells surrounded by WFA^+^ PNNs, **B** proportion of NeuN^+^ cells surrounded by WFA^+^ aggregates, **C** ratio between WFA^+^ PNNs and WFA^+^ aggregates, **D** proportion of PV^+^ cells surrounded by WFA^+^ PNNs, and **E** WFA^+^ PNNs surrounding PV^+^ cells. Data are presented as mean ± SD, *P*-values shown from RM one-way ANOVA. Note the significantly lower proportion of WFA^+^ PNNs, significantly higher proportion of WFA^+^ aggregates, and significantly lower degree of PV/WFA co-localization in BA24

WFA expression differed the most between the regions, with BA24 having a significantly lower total proportion of WFA^+^ PNNs (3.27 ± 0.69%) compared to BA9 (6.32 ± 1.73%; *P* = 0.0449, 95% CI 0.10 to 5.98, RM one-way ANOVA) and BA14r (5.64 ± 0.71%; *P* = 0.0278, 95% CI 0.39 to 4.34) (Fig. 8A). Inversely, BA24 had a significantly higher total proportion of WFA^+^ aggregates (2.10 ± 0.23%) compared to BA9 (1.54 ± 0.19%; *P* = 0.0183; 95% CI −0.96 to −0.15), while the difference compared to BA14r was not significant (1.63 ± 0.17%; *P* = 0.0553, 95% CI −0.95 to 0.02) (Fig. 8B). This difference between BA24 and the other analyzed regions is particularly apparent when plotting the ratio between the proportion of PNNs and the proportion of aggregates (Fig. 8C).

Even though the total proportions of NCAN^+^ and VCAN^+^ PNNs and aggregates did not differ significantly between the analyzed regions (Figure S4), we found subtle differences in the laminar expression of these markers. In particular, the total proportion of VCAN^+^ PNNs in layer VI was significantly higher in BA24 (4.45 ± 0.32%) than in BA9 (3.47 ± 0.61%; *P* = 0.0062, 95% CI −1.50 to −0.44), while the total proportion of NCAN^+^ PNNs in layer VI was significantly higher in BA24 (2.84 ± 0.36%) than in BA14r (1.84 ± 0.39%; *P* = 0.0213, 95% CI: −1.78 to −0.23).

We also analyzed the differences in PV/WFA co-localization and determined that the level of co-localization was generally lower in BA24 than in the other analyzed regions. In particular, the total proportion of PV^+^ neurons surrounded by WFA^+^ PNNs was significantly lower in BA24 (43.55 ± 4.67%) than in BA9 (58.92 ± 2.79%; *P* = 0.0013, 95% CI 9.20 to 19.54) and BA14r (51.90 ± 5.69%; *P* = 0.0079, 95% CI 3.48 to 13.21) (Fig. 8D). The total proportion of WFA^+^ PNNs surrounding PV^+^ neurons was also significantly lower in BA24 (37.04 ± 11.96%) than in BA9 (58.19 ± 4.80%; *P* = 0.0393, 95% CI 1.55 to 40.76), while the difference compared to BA14r was not significant (57.62 ± 9.30; *P* = 0.1388, 95% CI −8.99 to 50.17) (Fig. 8E). The statistical significance of the differences in the numerousness of WFA-, VCAN-, and NCAN-labeled PNNs between different layers within BA 9, 14r, and 24 of human PFC is provided in Supplementary material in Table S3.

In addition, we performed PV/WFA double labeling in the primary motor (M1) and primary sensory (S1) cortex of the human brain to explore differences in PNN distribution (Figure S6). M1 contained large PV^+^ neurons surrounded by well-developed WFA^+^ PNNs in the upper cortical layers (marked with white asterisks). The PV^+^ cells in the upper cortical layers in S1 were smaller and enveloped by less pronounced WFA^+^ PNNs. In the deep cortical layers in M1, large triangular WFA^+^ PNNs were present (marked with white arrows). These PNNs likely surrounded the giant pyramidal cells characteristically present in layer V of this cortical region.

### Transcriptomic Data Correlate Well with Histological Findings

**Fig. 9:**
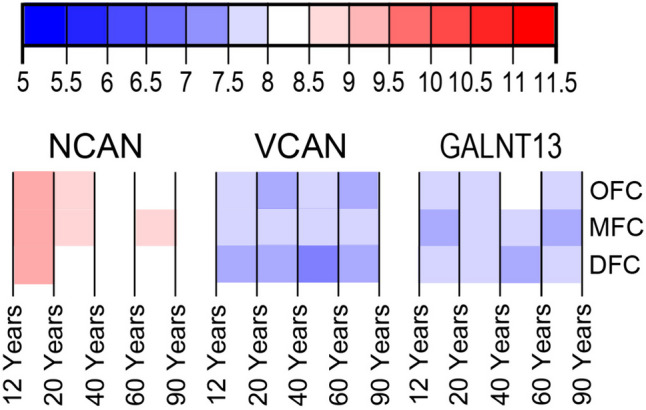
Heat map matrix displaying the log2-transformed signal intensity of *NCAN*, *VCAN*, and *GALNT13* expression across three regions (DFC, MFC, and OFC) during the period from 12 to 90 years of age. The heat map color scale ranges from low (blue) to high (red). *NCAN* had the highest expression (ranging from 8.0 to 9.5 log2-transformed signal intensity) during adolescence, decreasing the fastest in the DFC and slowest in the MFC. *VCAN* expression was relatively stable (from 6.5 to 8.0 log2-transformed signal intensity), with the highest expression in the MFC and the lowest in the DFC. *GALNT13* had the highest expression (from 7.0 to 8.5 log2-transformed signal intensity) out of all the *GALNT* genes (see Figure S5), particularly in the OFC

The analyzed transcript expression for *NCAN, VCAN*, and the GALNT family shows that these genes were expressed in all studied regions (DFC, MFC, OFC) during all examined lifespan periods (13–82 years). *NCAN* had the highest expression of all the analyzed genes, ranging from 8.0 to 9.5 log2-transformed signal intensity values during the examined period. *NCAN* transcript expression values gradually decrease during maturation, with the fastest decline in DFC and the slowest in MFC (Fig. 9). In contrast, *VCAN* expression had the highest values in the MFC and the lowest in the DFC, showing relatively stable expression levels over the examined period from 6.5 to 8.0 log2-transformed signal intensity (Fig. 9). Out of all the *GALNT* genes expressed in the brain (Figure S5), *GALNT13* had the highest expression (from 7.0 to 8.5), with the highest values in the OFC (Fig. 9). The *GALNT* transcripts show a relatively stable expression over the examined lifespan and either similar expression levels in all analyzed regions or a slight difference in expression between DFC and MFC (Figure S5).

## Discussion

This study explored the morphology, molecular characteristics, and spatial distribution of different ECM molecular markers in the adult human PFC. We determined the proportion of neurons enveloped by WFA^+^, NCAN^+^, and VCAN^+^ (V0 isoform) condensed ECM in Brodmann areas 9, 14r, and 24. The analysis of condensed ECM revealed two different types of cellular envelopment—ECM aggregates and PNNs. We examined the co-localization of ECM molecular constituents and different interneuron markers (PV, CR, SOM). In addition, we quantified the co-localization of PV^+^ cells and WFA^+^ PNNs. Finally, we assessed the regional and laminar distribution of the different types of condensed perisomatic ECM.

### WFA Labels a Specific Subset of PNNs Surrounding Subpopulations of PV^+^ Interneurons and Pyramidal Neurons

A major finding of our study is that WFA^+^ PNNs can be classified into at least three distinct morphological types. Each morphological type had a specific laminar distribution and surrounded different types of cells, suggesting that specific neuron populations are connected to particular morphological types of PNNs. We also observed that, in general, WFA^+^ PNNs had a higher staining intensity than most NCAN^+^ and VCAN^+^ PNNs, which was in correlation with the measured thickness of all PNN types. Only type 1 VCAN^+^ PNNs had a similar thickness to WFA^+^ PNNs. In addition, WFA^+^ PNNs were the only ones that could be traced beyond the first branching point of the cellular processes. This could be due to the fact that WFA binds to *N*-acetylgalactosamine found in the GAG side chains [65]. In contrast, anti-VCAN and anti-NCAN antibodies bind to the core protein region of the corresponding proteoglycans.

The ratio between the number of WFA^+^ PNNs and WFA^+^ aggregates was substantially higher than the corresponding ratios for NCAN and VCAN. This means that the number of WFA^+^ PNNs was much higher than that of WFA^+^ aggregates, which was not true for NCAN and VCAN. Once again, different binding sites of the analyzed ECM markers might explain some of these observed differences.

Even though there have been histological studies on WFA expression in primate brains [66–69], no studies so far seem to have quantified both PNNs and aggregates, nor have they provided clear methodological criteria for distinguishing these two types of condensed ECM.

Our analysis of the laminar distribution of WFA^+^ PNNs revealed a similar pattern described in previous research [24, 38]. However, we also found WFA^+^ PNNs sporadically in layer I, which has not been described so far. It is likely that using NeuN/WFA double labeling, we could distinguish between the cortical layers with a higher level of precision and more easily identify these relatively scarce WFA^+^ PNNs in layer I.

Quantitative analysis also confirmed that the total proportion of neurons surrounded by either WFA^+^ PNNs or WFA^+^ aggregates differed significantly in BA24 compared to BA9 and BA14r. This was also reflected in the difference in the degree of co-localization between PV^+^ neurons and WFA^+^ PNNs. The differences in co-localization degree were most pronounced in layers II and VI. These differences most likely reflect the different cytoarchitectonics in BA24, which is defined as an agranular cortical region, while BA9 and BA14r are granular and dysgranular regions, respectively. In addition, BA24 is anatomically part of the limbic system. It has functional connections different from BA9 and BA14r, which might also be reflected in this region’s distinct expression of WFA+ PNNs. Triangular WFA^+^ PNNs were only sporadically found in the deep cortical layers of S1, and they were much smaller in size than in M1. No WFA^+^ aggregates could be observed in S1, while in M1, they were observed only rarely, which contrasts with the large number of WFA^+^ aggregates observed in the ACC (BA24). Similar to BA9, BA14r, and BA24, in M1 and S1, only some PV^+^ cells were enveloped by WFA^+^ PNNs, and some WFA^+^ PNNs enveloped cells that did not express PV.

When evaluating the co-localization of WFA^+^ PNNs with different GABAergic interneuron markers, we found a significant degree of co-localization only with the PV^+^ neuron population, which agrees with previous research [24, 38].

The qualitative analysis of WFA, NCAN, and VCAN co-localization on triple-labeling slides revealed a relatively low co-localization. In addition, only NCAN and WFA showed any substantial degree of co-localization, mainly in layer III. From the analysis of PV/WFA and PV/NCAN double labeling, we can infer that the NCAN^+^/WFA^+^ PNNs are likely found surrounding PV^+^ neurons in layer III. This means layer III PV^+^ neurons seem surrounded by a specific molecular composition of PNNs, where NCAN is the major lectican, and N-acetylgalactosamine is the primary side GAG. In contrast, there was almost no co-localization between VCAN and WFA. This aligns with our findings on double-labeled slides where we combined WFA or VCAN with all interneuron markers. While WFA co-localized only with PV^+^ neurons, VCAN co-localized with CR^+^ and SOM^+^ but not PV^+^ neurons. This suggests that VCAN^+^ PNNs either have a different side GAG (not *N*-acetylgalactosamine) or that the quantity of *N*-acetylgalactosamine in these PNNs is below the detection threshold for WFA staining.

### VCAN (V0) Forms PNNs Surrounding Specific Subpopulations of CR and SOM Interneurons

Another significant finding of our study was that VCAN^+^ PNNs could, similar to WFA^+^ PNNs, also be classified into at least three distinct morphological types, which surrounded different types of cells and had specific laminar distributions. While type 3 WFA^+^ PNNs surrounding pyramidal cells were relatively abundant and most numerous in layer III, type 3 VCAN^+^ PNNs were found only sporadically and almost exclusively in layer V. The ratio between the number of VCAN^+^ PNNs and VCAN^+^ aggregates was higher than the corresponding NCAN ratio but lower than for WFA.

Unlike WFA^+^ PNNs, the highest proportion of neurons surrounded by VCAN^+^ PNNs were found in layers I and VI. Compared to WFA^+^ PNNs, regional differences were relatively subtle, with only the proportion of VCAN^+^ PNNs in layer VI being significantly different in BA24.

When evaluating the co-localization of VCAN^+^ PNNs with different GABAergic interneuron markers, we found co-localization with SOM^+^ neurons in layers V/VI and with CR^+^ neurons in layers I, III, and IV. At the same time, there was no co-localization with PV^+^ neurons. In addition, we observed a certain amount of PNNs, predominantly located in layers V and VI, that expressed both NCAN and VCAN. This finding is particularly interesting since it suggests that a single PNN could contain multiple types of lecticans. This concurs with our finding on SOM/NCAN and SOM/VCAN double labeling, where we observed both NCAN^+^ and VCAN^+^ PNNs surrounding SOM^+^ neurons in layers V and VI. We found no substantial co-expression of VCAN with WFA, which aligns with the finding that VCAN^+^ PNNs did not co-localize with PV^+^ neurons.

### NCAN Forms PNNs Surrounding All Significant GABAergic Interneuron Populations

NCAN^+^ PNNs mostly surrounded smaller circular neurons and were found in all cortical layers. The proportion of neurons surrounded by NCAN^+^ PNNs was the lowest of the three analyzed ECM markers. The ratio between the number of NCAN^+^ PNNs and NCAN^+^ aggregates was substantially lower than the corresponding ratios for WFA and VCAN. This means the number of NCAN^+^ PNNs was much lower than that of NCAN^+^ aggregates.

Similar to VCAN^+^ PNNs, the highest proportion of neurons surrounded by NCAN^+^ PNNs were found in layers I and VI. Regional differences between BA24, BA9, and BA14r were relatively subtle, with only the proportion of NCAN^+^ PNNs in layer VI being significantly different, similar to VCAN.

When evaluating the co-localization of NCAN^+^ PNNs with different GABAergic interneuron markers, we found co-localization with the PV^+^ neurons in layer III, with SOM^+^ neurons in layers V/VI, and with CR^+^ neurons in layers I and V/VI. NCAN^+^ PNNs were the only observed PNNs that surrounded all three major interneuron populations.

We identified WFA and NCAN co-localization in layer III, likely surrounding PV^+^ neurons using triple labeling. In contrast, the co-localization with VCAN was present in layers V and VI, possibly surrounding SOM^+^ neurons.

### Could Perisomatic ECM Aggregates Be a Sign of PNN Remodeling?

The finding of perisomatic ECM aggregates seems particularly noteworthy. Even though ECM aggregates represent a significant portion of condensed perisomatic ECM, their functional significance is challenging to elucidate. We propose several possibilities to explain the presence of ECM aggregates. One possibility could be viewing PNNs as dynamic structures that change due to episodes of neuronal plasticity in the adult brain [3]. In light of this, ECM aggregates could represent PNNs in different states of formation or degradation. This notion may be supported by the fact that research has already shown changes in PNNs due to enriched environmental stimulation [70, 71], sleep deprivation [72], or injury-induced remodeling [73–75]. However, it should be noted that most of the data on PNN remodeling is derived from animal models, and it is difficult to determine the translational potential of these studies. It could also be possible that condensed ECM in the brain undergoes regular remodeling during learning and aging, possibly influenced by metalloproteinase activity or changes in the synthesis of PNN components and chondroitin sulfate-GAG sulfation patterns [76–79].

### The Specific Laminar, Regional, and Perineuronal Expression of Different ECM Markers Could Give an Essential Insight into the Functional Organization of Specific Microcircuits

The presented findings contribute to the growing knowledge about the high specificity of ECM molecular constituents surrounding specific neuron populations in particular cortical regions and layers. This could imply that different microcircuits might be associated with distinct ECM expression patterns found in PNNs. Such an implication is potentially important when considering the amount of data showing rather selective intra- and extracellular alterations in various neuropsychiatric disorders or lesions of the CNS. For example, significant alterations of PNNs were found in the human PFC in bipolar disorder, major depression disorder, and schizophrenia [23] as well as in the human PFC, occipital, and temporal cortices in Alzheimer’s disease [37, 39]. In addition, animal models of different types of ischemia showed evidence of PNN remodeling occurring in the rodent cortex [25, 27, 80–82]. There is also some evidence of ECM reorganization in epilepsy in humans [16, 41] as well as in mouse models of binge drinking [83] and juvenile stress [84]. If such alterations indeed underlie the pathogenesis of these disorders, it would be especially beneficial to determine which cell populations are exactly affected. In addition, there is growing evidence that certain neuropathological disorders are connected to abnormal glycosylation in general [85, 86]. It would be particularly interesting to determine whether this translates to any alterations in the structure of PNNs. Therefore, more research is needed on the expression of different neurons and ECM markers in various regions of the brain to determine which exact microcircuits might be affected by neuropathology.

Our study is the first to give a more comprehensive description of the expression of different ECM markers across all cortical layers and in different cortical regions of the human brain. We also provided a solid basis for morphological and molecular classification of ECM in the human cerebral cortex. So far, the only classification of ECM seems to have been made in the rat olfactory bulb [87]. We also found no studies evaluating the expression of VCAN and NCAN in the human cerebral cortex. We focused primarily on the different parts of the PFC, which is considered to be especially important in regulating higher cognitive functions. Many neuropathological disorders are also related to the dysfunction of the PFC and its connectivity to other regions of the brain [23, 88, 89]. We chose different cortical regions for analysis, all part of the prefrontal cortex (PFC) specific for human highest cognitive functions, but still belonging to three different types of cortical cytoarchitectonics: the dorsolateral PFC (BA9 granular neocortex), ventromedial (vm) PFC (BA14r dysgranular cortex), and anterior subgenual cingulate cortex (BA24, agranular) which imply developmental and evolutional diversity. Therefore, all the PNN differences we found between these areas could potentially be related to microcircuitry organization and functional differences, as well as cytoarchitectonic variants in the broader context of cortical organization. In addition, found varieties may imply that these differences could be even more significant in more cytoarchitectonically and functionally distant cortical areas. Therefore, our study provides critical normative data and grounds future research on ECM alterations in neuropathological conditions. Such research may significantly benefit from focusing on different types of ECM molecules and interneuron populations instead of focusing solely on PV neurons and their co-localization with WFA.

### Lifespan Transcriptome Shows Only Slight Differential Expression Between Regions, Relatively Stable Levels of PNN-Related Transcripts During Adulthood, and Slow Expression Decline with Aging

The anatomical-histological and transcriptomic data are needed to understand the presented findings fully. The immunohistochemical data gives us the precise location of proteins analyzed in this study. However, the presence of proteins in a specific area does not necessarily mean that the proteins are synthesized in these areas; they could be synthesized in distant locations and transported to a particular area. The presented transcriptomic data signifies that the analyzed proteins are synthesized locally in the analyzed areas. The transcriptomic analysis of *VCAN* and *NCAN* revealed medium to high expression levels in all cortical regions during the entire lifespan. There were no substantial differences between the cortical regions, which is in line with the results of the histological analysis. Overall, *NCAN* expression was slightly higher than that of *VCAN*. This also seems to be in line with the histological findings. At the same time, NCAN^+^ PNNs were slightly scarcer, NCAN^+^ aggregates were more abundant, and qualitative observations indicated that diffuse NCAN seemed to be more prevalent than diffuse VCAN on histological slides.

The connection between the expression of *GALNT* transcripts and WFA staining on histological slides was less pronounced. This could be explained by the fact that, unlike the direct relation between the transcripts of lecticans and their protein products, the relation between the transcript (an enzyme) and the final product (WFA binding to GAG side chains) is indirect. Nevertheless, the transcriptomic analysis revealed that GALNT13 had the highest expression among the GALNT family, making it a good potential target for further studies on glycosylation patterns in the human cortex.

## Conclusions

In conclusion, perisomatic condensation of ECM in the adult human PFC exhibits a high level of molecular diversity. Each ECM molecular marker we analyzed (WFA, NCAN, and VCAN) formed two types of condensed ECM—PNNs and aggregates. These two forms of condensed ECM could be distinguished based on the degree of distinction between the signal and background and on the degree to which they envelop cellular processes. The ratio between PNNs and ECM aggregates differed substantially between different markers, with WFA staining predominantly visualizing PNNs and NCAN staining predominantly visualizing aggregates. Each ECM marker had a characteristic laminar distribution, with WFA^+^ PNNs being most numerous in layers III, IV, and V and NCAN^+^ and VCAN^+^ PNNs being most numerous in layers I and VI. Regional differences were most pronounced in WFA staining, where BA24 differed significantly from BA9 and BA14r in the total proportion of WFA^+^ PNNs and aggregates. Finally, the co-localization with different interneuron markers (PV, CR, and SOM) was highly specific for each ECM marker. The presented data point towards a high degree of ECM specialization, possibly related to different cortical microcircuits. Understanding the basis of such specialization is of great importance for determining the possible significance of ECM alterations in various neuropsychiatric conditions, such as schizophrenia, bipolar disorder, and major depressive disorder.

## Supplementary Information

ESM 1 (TIF 50415 kb)

ESM 2 (TIF 61300 kb)

ESM 3 (TIF 6029 kb)

ESM 4 (TIF 4200 kb)

ESM 5 (TIF 7499 kb)

ESM 6 (TIF 172275 kb)

ESM 7 (ZIP 168999 kb)

ESM 8 (DOCX 27 kb)

## Data Availability

All data presented in this study are available in the article and accompanying supplementary information. Raw data are available on request from the corresponding author.
