# Supplementary figures and images for: Morphological and Molecular Characteristics of Perineuronal Nets in the Human Prefrontal Cortex—A Possible Link to Microcircuitry Specialization
Source: Mol Neurobiol. 2024 Jul 3;62(1):1094–111. doi: 10.1007/s12035-024-04306-1 (PMC11711633; doi:10.1007/s12035-024-04306-1)

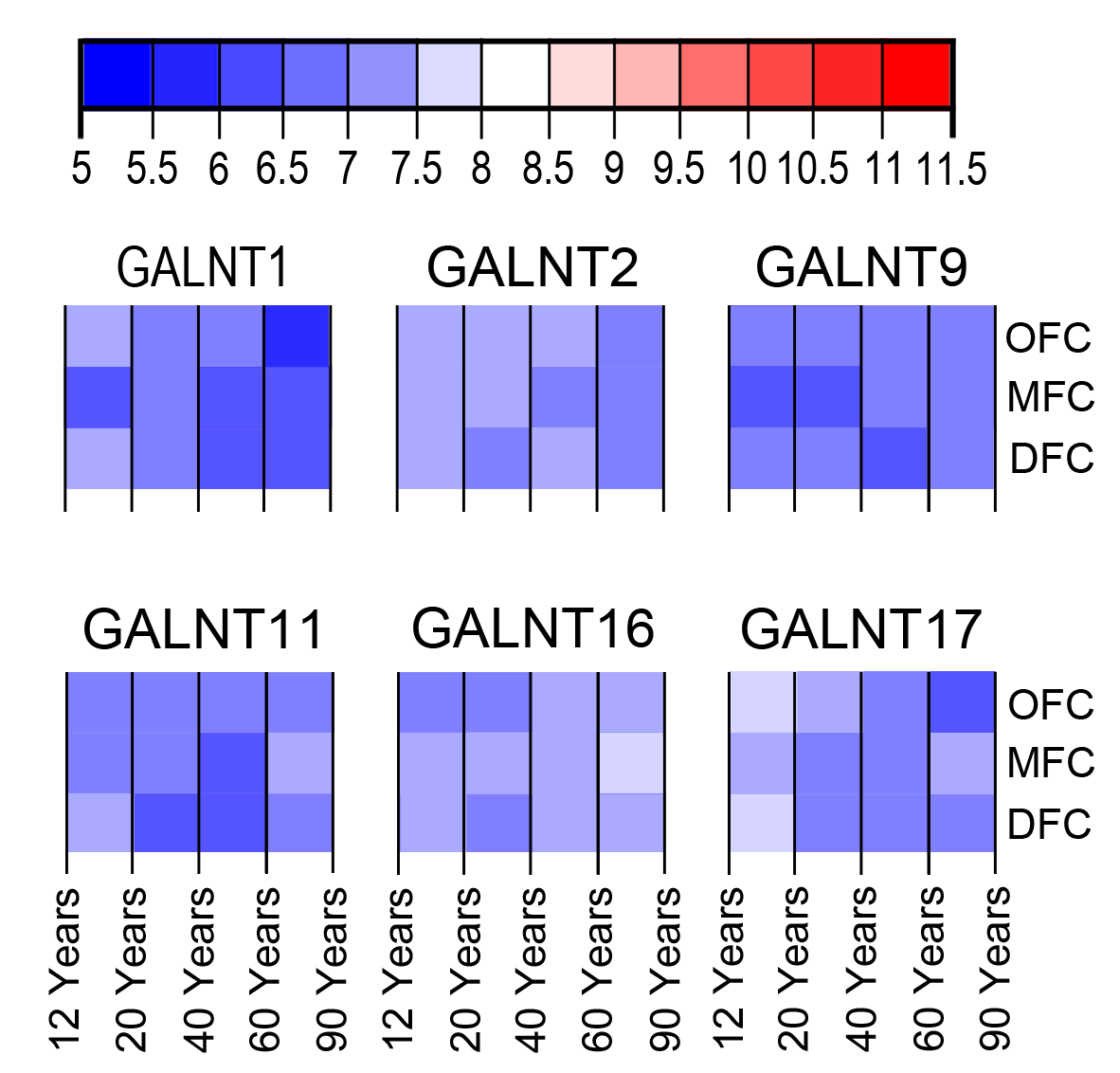

Supplement: Supplementary file 5 — (TIF 7499 kb) [file 12035_2024_4306_MOESM5_ESM.tif]
