# Supplementary material for: Morphological and Molecular Characteristics of Perineuronal Nets in the Human Prefrontal Cortex—A Possible Link to Microcircuitry Specialization
Source: Mol Neurobiol. 2024 Jul 3;62(1):1094–111. doi: 10.1007/s12035-024-04306-1 (PMC11711633; doi:10.1007/s12035-024-04306-1)
Supplement: Supplementary file 8 — (DOCX 27 kb) [file 12035_2024_4306_MOESM8_ESM.docx]

Tables

Table S1. Human brain tissue used for immunofluorescence and transcriptomics.

| **Subject** | **Sex** | **Age (years)** | **Postmortem interval (h)** | **Cause of death** | **Methodology** |
| --- | --- | --- | --- | --- | --- |
| CO382 | M | 40 | 6.5 | sudden cardiac death | immunofluorescence |
| CO383 | M | 51 | 11 | sudden cardiac death | immunofluorescence |
| CO384 | M | 46 | 6 | sudden cardiac death | immunofluorescence |
| CO386 | M | 37 | 6 | sudden cardiac death | immunofluorescence |
| CO387 | M | 44 | 6 | methadone/benzodiazepine overdose | immunofluorescence |
| HSB124 | F | 13 | 19.5 | homicide | transcriptomics |
| HSB119 | M | 15 | 14.5 | homicide | transcriptomics |
| HSB105 | M | 18 | 28 | heart failure | transcriptomics |
| HSB127 | F | 19 | 9.5 | heart failure | transcriptomics |
| HSB130 | F | 21 | 18 | anaphylaxis | transcriptomics |
| HSB142 | M | 22 | 18 | heart failure | transcriptomics |
| HSB136 | M | 23 | 10.5 | homicide | transcriptomics |
| HSB133 | F | 27 | 21 | heart failure | transcriptomics |
| HSB125 | M | 28 | 7.5 | heart failure | transcriptomics |
| HSB126 | F | 30 | 9.5 | heart failure | transcriptomics |
| HSB145 | M | 36 | 18 | heart failure | transcriptomics |
| HSB123 | M | 37 | 13 | subarachnoid hemorrhage | transcriptomics |
| HSB187 | F | 37 | 10 | heart failure | transcriptomics |
| HSB144 | M | 40 | 28 | heart failure | transcriptomics |
| HSB135 | F | 40 | 30.5 | heart failure | transcriptomics |
| HSB183 | M | 42 | 19 | heart failure | transcriptomics |
| HSB182 | M | 55 | 13 | heart failure | transcriptomics |
| HSB106 | M | 64 | 4 | undetermined | transcriptomics |
| HSB111 | F | 70 | 13 | aortic dissection | transcriptomics |
| HSB156 | F | 82 | 16 | pulmonary embolism | transcriptomics |

Table S2. Primary and secondary antibodies used for immunofluorescence.

| **Antibody** | **Species and clonality** | **Manufacturer, catalogue number (CN), lot** | **Working dilution** |
| --- | --- | --- | --- |
| **Primary antibodies** | | | |
| anti-calretinin | mouse; monoclonal | Swant;  CN: 6B3;  lot: 010399 | 1:1000 |
| anti-parvalbumin | rabbit; polyclonal | abcam;  CN: ab11427;  lot: GR3317380-2 | 1:1000 |
| anti-parvalbumin (PARV-19) | mouse; monoclonal | Sigma-Aldrich;  CN: MAB1572;  lot: 128M4891V | 1:4000 |
| anti-calbindin | rabbit; polyclonal | Swant;  CN: CB-38a;  lot: 9.03 | 1:10000 |
| anti-somatostatin-14 | rabbit; polyclonal | Penninsula;  CN: T-4101.0500;  lot: A1819 | 1:4000 |
| anti-somatostatin (7G5) | mouse; monoclonal | ThermoFisher (Invitrogen);  CN: MA5-17182  lot: VK3133146 | 1:2000 |
| anti-NeuN | rabbit; polyclonal | abcam;  CN: ab104225;  lot: GR3410699-1 | 1:1000 |
| anti-NeuN (1B7) | mouse; monoclonal | abcam;  CN: ab104224;  lot: 1014680-1 | 1:1000 |
| anti-NCAN | rabbit; polyclonal | Sigma-Aldrich;  CN: HPA036814  lot: A 107045 | 1:1000 |
| anti-VCAN V0 | goat; polyclonal | Novus Biologicals;  CN: AF3054;  lot: P13611 | 1:2000 |
| WFA , Fluorescein | N/A | Vector Laboratories;  CN: FL-1351;  lot: ZG0903 | 1:200 |
| **Secondary antibodies** | | | |
| conjugated anti-mouse Alexa 488 | donkey | ThermoFisher (Invitrogen);  CN: A21202;  lot: 2428531 | 1:1000 |
| conjugated anti-mouse Alexa 555 | donkey | ThermoFisher (Invitrogen);  CN: A31570;  lot: 2253917 | 1:1000 |
| conjugated anti-rabbit Alexa 546 | donkey | ThermoFisher (Invitrogen);  CN: A10040;  lot: 2128963 | 1:1000 |
| conjugated anti-goat Alexa 488 | donkey | ThermoFisher (Invitrogen);  CN: A32814;  lot: VA293145 | 1:1000 |
| conjugated anti-goat Alexa 647 | donkey | ThermoFisher (Invitrogen);  CN: A21447;  lot: 1917928 | 1:1000 |
| conjugated anti-mouse Alexa 647 | goat | ThermoFisher (Invitrogen);  CN: A32728;  lot: UK290265 | 1:1000 |

Table S3. The significance of the quantitative differences in numerousness of the WFA, VCAN, and NCAN labeled PNNs between different layers in BA 9, 14r, and 24 of human PFC (** - p values ≤ 0.005).

WFA:

| Comparison | Mean Diff, | 95,00% CI of diff, | Below threshold? | Summary | Adjusted P Value |
| --- | --- | --- | --- | --- | --- |
| III 9 vs. I 9 | 10,40 | 0,1498 to 20,65 | Yes | * | 0,0476 |
| IV 9 vs. I 9 | 6,630 | 0,1506 to 13,11 | Yes | * | 0,0462 |
| V 9 vs. I 9 | 7,378 | 2,373 to 12,38 | Yes | * | 0,0124 |
| V 9 vs. II 9 | 6,078 | 1,030 to 11,13 | Yes | * | 0,0260 |
| VI 9 vs. I 9 | 2,852 | 0,7965 to 4,907 | Yes | * | 0,0155 |
| III 14 vs. I 14 | 7,872 | 4,408 to 11,34 | Yes | ** | 0,0025 |
| III 14 vs. II 14 | 7,264 | 2,457 to 12,07 | Yes | * | 0,0113 |
| V 14 vs. I 14 | 5,606 | 1,640 to 9,572 | Yes | * | 0,0144 |
| V 14 vs. II 14 | 4,998 | 1,105 to 8,891 | Yes | * | 0,0206 |
| VI 14 vs. III 14 | -5,644 | -11,23 to -0,05975 | Yes | * | 0,0482 |
| VI 14 vs. V 14 | -3,378 | -6,148 to -0,6076 | Yes | * | 0,0248 |
| III 24 vs. I 24 | 8,144 | 2,568 to 13,72 | Yes | * | 0,0128 |
| III 24 vs. II 24 | 7,362 | 3,482 to 11,24 | Yes | ** | 0,0047 |
| V 24 vs. I 24 | 5,394 | 1,847 to 8,941 | Yes | * | 0,0110 |
| V 24 vs. II 24 | 4,612 | 2,725 to 6,499 | Yes | ** | 0,0018 |
| VI 24 vs. III 24 | -6,048 | -8,541 to -3,555 | Yes | ** | 0,0019 |
|  |  |  |  |  |  |

VCAN:

| Comparison | Mean Diff, | 95,00% CI of diff, | Below threshold? | Summary | Adjusted P Value |
| --- | --- | --- | --- | --- | --- |
| VI 9 vs. II 9 | 2,342 | 0,6390 to 4,045 | Yes | * | 0,0160 |
| VI 9 vs. III 9 | 2,150 | 0,09922 to 4,201 | Yes | * | 0,0424 |
| VI 9 vs. IV 9 | 1,938 | 0,2894 to 3,587 | Yes | * | 0,0283 |
| II 14 vs. I 14 | -1,818 | -3,615 to -0,02135 | Yes | * | 0,0480 |
| V 14 vs. I 14 | -1,884 | -3,546 to -0,2216 | Yes | * | 0,0323 |
| VI 14 vs. II 14 | 2,950 | 1,023 to 4,877 | Yes | * | 0,0107 |
| VI 14 vs. III 14 | 2,752 | 0,04390 to 5,460 | Yes | * | 0,0473 |
| VI 24 vs. II 24 | 2,530 | 0,8699 to 4,190 | Yes | * | 0,0109 |

NCAN:

| Comparison | Mean Diff, | 95,00% CI of diff, | Below threshold? | Summary | Adjusted P Value |
| --- | --- | --- | --- | --- | --- |
| II 9 vs. I 9 | -1,988 | -3,464 to -0,5122 | Yes | * | 0,0173 |
| VI 24 vs. II 24 | 2,364 | 1,359 to 3,369 | Yes | ** | 0,0022 |
